# Kernel Entropy Component Analysis with Nongreedy L1-Norm Maximization

**DOI:** 10.1155/2018/6791683

**Published:** 2018-10-14

**Authors:** Haijin Ji, Song Huang

**Affiliations:** ^1^Command & Control Engineering College, Army Engineering University of PLA, Nanjing 210007, China; ^2^School of Computer Science and Technology, Huaiyin Normal University, Huaian 223300, China

## Abstract

Kernel entropy component analysis (KECA) is a newly proposed dimensionality reduction (DR) method, which has showed superiority in many pattern analysis issues previously solved by principal component analysis (PCA). The optimized KECA (OKECA) is a state-of-the-art variant of KECA and can return projections retaining more expressive power than KECA. However, OKECA is sensitive to outliers and accused of its high computational complexities due to its inherent properties of L2-norm. To handle these two problems, we develop a new extension to KECA, namely, KECA-L1, for DR or feature extraction. KECA-L1 aims to find a more robust kernel decomposition matrix such that the extracted features retain information potential as much as possible, which is measured by L1-norm. Accordingly, we design a nongreedy iterative algorithm which has much faster convergence than OKECA's. Moreover, a general semisupervised classifier is developed for KECA-based methods and employed into the data classification. Extensive experiments on data classification and software defect prediction demonstrate that our new method is superior to most existing KECA- and PCA-based approaches. Code has been also made publicly available.

## 1. Introduction

Curse of dimensionality is one of the major issues in machine learning and pattern recognition [1]. It has motivated many scholars from different areas to properly implement dimensionality reduction (DR) to simplify the input space without degrading performances of learning algorithms. Various efficient methods associated with DR have been developed, such as independent component analysis (ICA) [2], linear discriminant analysis [3], principal component analysis (PCA) [4], projection pursuit [5], to name a few. Among these robust algorithms, PCA has been one of the most used techniques to perform feature extraction (or DR). PCA implements linear data transformation according to the projection matrix, which aims to maximize the second-order statistics of input datasets [6]. To extend PCA to nonlinear space, Schölkopf et al. [7] proposed the kernel PCA, the so-called KPCA method. The key of KPCA is to find the nonlinear relation between the input data and the kernel feature space (KFS) using the kernel matrix, which is derived from a positive semidefinite kernel function of computing inner products. Both PCA and KPCA perform data transformation by selecting the eigenvectors corresponding to the top eigenvalues of the projection matrix and the kernel matrix, respectively. All of them (including their variants) have experienced great success in different areas [8–12], such as image reconstruction [13], face recognition [14–17], image processing [18, 19], to name a few. However, as suggested by Zhang and Hancock [20], the DR should be performed according to the perspective of information theory for obtaining more acceptable results.

To improve performances of the aforementioned approaches to DR, Jessen [6] developed a new and completely different data transformation algorithm, namely, kernel entropy component analysis (KECA). The main difference between KECA and PCA or KPCA is that the optimal eigenvectors (or called entropic components) derived from KECA can compress the most Renyi entropy of the input data instead of being associated with top eigenvalues. The procedure of selecting the eigenvectors related to the Renyi entropy of the input space is started with a Parzen window kernel-based estimator [21]. Then, only the eigenvectors corresponding to the most entropy of the input datasets are selected to perform DR. This distinguished characteristic helps KECA achieve better performances than the classical PCA and KPCA in face recognition and clustering [6]. In recent years, Izquierdo-Verdiguier et al. [21] employed the rotation matrix from ICA [2] to optimize KECA and proposed the optimized KECA (OKECA). OKECA not only shows superiority in classification of both synthetic and real datasets but can obtain acceptable kernel density estimation (KDE) just using very fewer entropic components (just one or two) compared with KECA [21]. However, OKECA is sensitive to outliers for its inherent properties of L2-norm. In other words, if the input space follows normal distribution and is contaminated by nonnormal distributed outliers, this may lead to the downgrade of its performance on DR in terms of OKECA. Additionally, OKECA is very time-consuming when handling large-scale input datasets (Section 4).

Therefore, the main purpose of this paper is to propose a new variant of KECA and improve the proneness to outliers and efficiency of OKECA. L1-norm is well known for its robustness to outliers [22]. Additionally, Nie et al. [23] established a fast iteration process to handle the general L1-norm maximization issue with nongreedy algorithm. Hence, we take advantages of OKECA and propose a new L1-norm version of KECA (denoted as KECA-L1). KECA-L1 uses an efficient convergence procedure, motivated by Nie et al.'s method [23], to search for the entropic components contributing to the most Renyi entropy of input data. To evaluate the efficiency and effectiveness of KECA-L1, we design and conduct a series of experiments, in which the data vary from single class to multiattribute and from small to large size. The classical KECA and OKECA are also included for comparison.

The remainder of this paper is organized as follows: Section 2 reviews the general L1-norm maximization issue, KECA, and OKECA.  Section 3 presents KECA with nongreedy L1-norm maximization and semisupervised-learning-based classifier. Section 4 validates the performance of the new method on different data sets.  Section 5 ends this paper with some conclusions.

## 2. Preliminaries

### 2.1. An Efficient Algorithm to Solving the General L1-Norm Maximization Issue

The general L1-norm maximization problem is first raised by Nie et al. [23]. This issue, based on a hypothesis that there exists an upper bound for the objective function, can be generally formulated as [23]

(1) $$\begin{array}{c} \underset{\nu \in C}{\max}f\left(\nu \right)+\underset{i}{\sum }\left|g_{i}\left(\nu \right)\right|, \end{array}$$

where both *f*(*ν*) and *g*_*i*_(*ν*) for each *i* denote arbitrary functions, and *ν* ∈ *𝒞* represents an arbitrary constraint.

Then a sign function sign(·) is defined as

(2) $$\begin{array}{c} \mathrm{sign}\left(x\right)=\left\{\begin{array}{cc} 1 & \text{if}\;\;x\geq 0,\\ -1 & \text{if}\;\;x<0, \end{array}\right. \end{array}$$

and employed to transform the maximization problem (1) as follows:

(3) $$\begin{array}{c} \underset{\nu \in C}{\max}f\left(\nu \right)+\underset{i}{\sum }\alpha_{i}g_{i}\left(\nu \right), \end{array}$$

where *α*_*i*_=sign(*g*_*i*_(*ν*)). Nie et al. [23] proposed a fast iteration process to solve problem (3), which is shown in Algorithm 1. It can be seen from Algorithm 1 that *α*_*i*_ is determined by current solution *ν* ^*t*^, and the next solution *ν* ^*t*+1^ is updated according to the current *α*_*i*_. The iterative process is repeated until the procedure converges [23, 24]. The convergence of the Algorithm 1 has been demonstrated, and the associated details can also be read in [23].

### 2.2. Kernel Entropy Component Analysis

KECA is characterized by its entropic components instead of the principal or variance-based components in PCA or KPCA, respectively. Hence, we firstly describe the concept of the Renyi quadratic entropy. Given the input dataset **X**=[**x**_1_,…, **x**_*N*_](**x**_*i*_ ∈ *ℝ* ^*D*^), the Renyi entropy of **X** is defined as [6]

(4) $$\begin{array}{c} H\left(p\right)=-\log\int p^{2}\left(x\right)dx,\;x\in X, \end{array}$$

where *p*(**x**) is a probability density function. Based on the monotonic property of logarithmic function, Equation (4) can be rewritten as

(5) $$\begin{array}{c} V\left(p\right)=\int p^{2}\left(x\right)dx. \end{array}$$

We can estimate Equation (5) using the kernel *k*_*σ*_(**x**, **x**_*t*_) of Parzen window density estimator determined by the bandwidth coefficient *σ* [6] such that

(6) $$\begin{array}{c} V\left(p\right)\approx \hat{V}\left(p\right)\\ =\frac{1}{N}\underset{x\in X}{\sum }p\left(x\right)\\ =\frac{1}{N}\underset{x_{i}\in X}{\sum }\frac{1}{N}\underset{x_{j}\in X}{\sum }k_{\sigma }\left(x_{i},x_{j}\right)\\ =\frac{1}{N^{2}}1^{T}K1, \end{array}$$

where **K**_*ij*_=*k*_*σ*_(**x**_*i*_, **x**_*j*_) constitutes the kernel matrix **K** and **1** represents an *N*-dimensional vector containing all ones. With the help of the kernel decomposition [6],

(7) $$\begin{array}{c} K=AA^{T}=\left(ED^{1/2}\right)\left(D^{1/2}E^{T}\right). \end{array}$$

Equation (6) is transformed as follows:

(8) $$\begin{array}{c} \hat{V}\left(p\right)=\frac{1}{N^{2}}\overset{N}{\underset{i=1}{\sum }}{\left(\sqrt{\lambda_{i}}1^{T}e_{i}\right)}^{2}, \end{array}$$

where the diagonal matrix **D** and the matrix **E** consist of eigenvalues *λ*_1_,…, *λ*_*N*_ and the corresponding eigenvectors **e**_1_,…, **e**_*N*_, respectively. It can be observed from Equation (7) that the entropy estimator $\hat{V}\left(p\right)$ consists of projections onto all the KFS axes because

(9) $$\begin{array}{c} K_{ij}=k_{\sigma }\left(x_{i},x_{j}\right)=\phi {\left(x_{i}\right)}^{T}\phi \left(x_{j}\right), \end{array}$$

where the function of *ϕ*(·) is to map the two samples **x**_*i*_ and **x**_*j*_ into the KFS. Additionally, only an entropic component **e**_*i*_ meeting the criteria of *λ*_*i*_ ≠ 0 and **1** ^*T*^ **e**_*i*_ ≠ 0 can contribute to the entropy estimate [21]. In a word, KECA implements DR by projecting *ϕ*(**X**) into a subspace **E**_*l*_ spanned not by the eigenvectors associated with the top eigenvalues but by entropic components contributing most to the Renyi entropy estimator $\hat{V}\left(p\right)$ [25].

### 2.3. Optimized Kernel Entropy Component Analysis

Due to the fact that KECA is sensitive to different bandwidth coefficients *σ* [21], OKECA is proposed to fill this gap and improve performances of KECA on DR. Motivated by the fast ICA method [2], an extra rotation matrix (applying **W**) is employed to the kernel decomposition (Equation (7)) in KECA for maximizing the information potential (the entropy values in Equation (8)) [21]:

(10) $$\begin{array}{c} \;\left\{\begin{array}{c} \underset{w_{k}\in W}{\max}\;J\left(w\right)={\left(1_{N}^{T}ED^{1/2}w\right)}^{2},\\ \text{s.t.}\;\;\;\;\;WW^{T}=I,\;\;{\left\|w\right\|}_{2}=1, \end{array}\right. \end{array}$$

where ‖·‖_2_ is the L2-norm and **w** denotes a column vector (*N* × 1) in **W**. Izquierdo-Verdiguier et al. [21] utilized a gradient-ascent approach to handle the maximization problem (10):

(11) $$\begin{array}{c} w\left(t\right)=w\left(t-1\right)+\tau \frac{\partial J}{\partial w\left(t\right)}, \end{array}$$

where *τ* is the step size. ∂*J*/∂**w**(*t*) can be obtained by Lagrangian multiplier:

(12) $$\begin{array}{c} \frac{\partial J}{\partial w\left(t\right)}=\frac{\partial ℒ\left(w\right)}{\partial w}=2\left(1_{N}^{T}ED^{1/2}w\right){\left(1_{N}^{T}ED^{1/2}\right)}^{T}. \end{array}$$

The entropic components multiplied by the rotation matrix can obtain more (or equal) information potential than that of the KECA even using fewer components [21]. Moreover, OKECA shows the capability of being robust to the bandwidth coefficient. However, there exist two main limitations for OKECA. First, the new entropic components derived from OKECA are sensible to outliers since its inherent properties of L2-norm (Equation (10)). Second, although a very simple stopping criterion is designed to avoid additional iterations, OKECA is still of high computational complexities for its computational cost is *O*(*N* ^3^+4*tN* ^2^) [21], where *t* is the number of iterations for finding the optimal rotation matrix, compared with that the one of KECA is *O*(*N* ^3^) [21].

## 3. KECA with Nongreedy L1-Norm Maximization

### 3.1. Algorithm

In order to alleviate the problems existing in OKECA, this section presents how to extend KECA to its nongreedy L1-norm version. For readers' easy understanding, the definition of L1-norm is firstly introduced as follows:

*Definition 1*. Given an arbitrary vector **x** ∈ *ℝ* ^*N*×1^, the L1-norm of the vector **x** is

(13) $$\begin{array}{c} {\left\|x\right\|}_{1}=\overset{N}{\underset{j=1}{\sum }}\left|x_{j}\right|, \end{array}$$

where ‖·‖_1_ is the L1-norm and *x*_*j*_ denotes the *j*th element of **x**.

Then, motivated by OKECA, we attempt to develop a new objective function to maximize the information potential (Equations (8) and (10)) based on the L1-norm:

(14) $$\begin{array}{c} \left\{\begin{array}{c} \max\;J\left(W\right)={\left\|W^{T}ED^{1/2}\right\|}_{1}=\overset{N}{\underset{j=1}{\sum }}\mathrm{sign}\left(a_{j}^{T}W\right)W^{T}a_{j},\\ \text{s.t.}\;\;\;\;\;W^{T}W=I, \end{array}\right. \end{array}$$

where (**a**_1_,…, **a**_*N*_)=**A**=**E** **D** ^1/2^, *N* is the size of samples. The rotation matrix is denoted as **W** ∈ *ℝ* ^DIM×*m*^, where DIM and *m* are the dimension of input data and dimension of the selected entropic components (or number of projection), respectively. It is difficult to directly solve problem (14), but we may regard it as a special case of problem (1) when *f*(*ν*) ≡ 0. Therefore, the Algorithm 1 can be employed to solve (14). Next, we show the details about how to find the optimal solution of problem (14) based on the proposal from References [23, 24]. Let

(15) $$\begin{array}{c} M=\overset{N}{\underset{j=1}{\sum }}a_{j}\mathrm{sign}\left(a_{j}^{T}W\right). \end{array}$$

Thus, problem (14) can be simplified as

(16) $$\begin{array}{c} \underset{W^{T}W=I}{\max}\;J\left(w\right)=\text{Tr}\left(W^{T}M\right). \end{array}$$

By singular value decomposition (SVD), then

(17) $$\begin{array}{c} M=U\Lambda V^{T}, \end{array}$$

where **U** ∈ *ℝ* ^DIM×DIM^, Λ ∈ *ℝ* ^DIM×*m*^, and **V** ∈ *ℝ* ^*m*×*m*^. Then we obtain

(18) $$\begin{array}{c} \text{Tr}\left(W^{T}M\right)=\text{Tr}\left(W^{T}U\Lambda V^{T}\right)=\text{Tr}\left(\Lambda V^{T}W^{T}U\right)\\ \;\;\;\;\;\;=\text{Tr}\left(\Lambda Z\right)=\underset{i}{\sum }\lambda_{ii}z_{ii}, \end{array}$$

where **Z** ∈ *ℝ* ^*m*×*m*^, *λ*_*ii*_ and *z*_*ii*_ denote the (*i*, *i*) − th element of matrix Λ and **Z**, respectively. Due to the property of SVD, we have *λ*_*ii*_ ≥ 0. Additionally, **Z** is an orthonormal matrix [23] such that *z*_*ii*_ ≤ 1. Therefore, Tr(**W** ^*T*^ **M**) can reach the maximum only if **Z**=[**I**_*m*_, **0**_*m*×(DIM − *m*)_], where **I**_*m*_ denotes the *m* × *m* identity matrix, and **0**_*m*×(DIM − *m*)_ is a *m* × (DIM − *m*) matrix of zeros. Considering that **Z**=**V** ^*T*^ **W** ^*T*^ **U**, thus the solution to problem (16) is

(19) $$\begin{array}{c} W=U\left[I_{m};0_{\left(\text{DIM}-m\right)\times m}\right]V^{T}. \end{array}$$

Algorithm 2 (A MATLAB implementation of the algorithm is available at the Supporting Document for the interested readers) shows how to utilize the nongreedy L1-norm maximization described in Algorithm 1 to compute Equation (19). Since problem (16) is a special case of problem (1), we can obviously obtain that the optimal solution **W** ^*∗*^ to Equation (19) is a local maximum point for ‖**W** ^*T*^ **E** **D** ^1/2^‖_1_ based on Theorem 2 in Reference [23]. Moreover, the Phase 1 of the Algorithm 2 spends *O*(*N* ^3^) on the eigen decomposition. Thus, the total of computational cost of KECA-L1 is *O*(*N* ^3^+*Nt*), where *t* is the number of iterations for convergence. Considering that the computational complexity of OKECA is *O*(*N* ^3^+4*tN* ^2^), we can safely conclude that KECA-L1 has much faster convergence than OKECA's.

### 3.2. The Convergence Analysis

This subsection attempts to demonstrate the convergence of the Algorithm 2 in the following: theorem:

**Theorem 1 .**

The above KECA-L1 procedure can converge.

**Proof**

Motivated by References [23, 24], first we show the objective function (9) of KECA-L1 will monotonically increase in each iteration *t*. Let *g*_*i*_(*u* ^*t*^)=**W** ^*T*^ **a**_*j*_ and *α*_*i*_^*t*^=sign(**a**_*j*_^*T*^ **W**), then (9) can be simplified to

(20) $$\begin{array}{c} \left\{\begin{array}{c} \max\;J\left(W\right)=\overset{N}{\underset{j=1}{\sum }}\mathrm{sign}\left(a_{j}^{T}W\right)W^{T}a_{j}=\overset{N}{\underset{j=1}{\sum }}\alpha_{i}^{t}g_{i}\left(u^{t}\right),\\ \text{s.t.}\;\;W^{T}W=I. \end{array}\right. \end{array}$$

Obviously, *α*_*i*_^*t*+1^ is parallel to *g*_*i*_(*u* ^*t*+1^), but neither is *α*_*i*_^*t*^. Therefore,

(21) $$\begin{array}{c} \left|g_{i}\left(u^{t+1}\right)\right|=\alpha_{i}^{t+1}g_{i}\left(u^{t+1}\right)\geq \alpha_{i}^{t}g_{i}\left(u^{t+1}\right)\Rightarrow \left|g_{i}\left(u^{t+1}\right)\right|-\alpha_{i}^{t}g_{i}\left(u^{t+1}\right)\geq 0. \end{array}$$

Considering that |*g*_*i*_(*u* ^*t*^)|=*α*_*i*_^*t*^ *g*_*i*_(*u* ^*t*^), thus

(22) $$\begin{array}{c} \left|g_{i}\left(u^{t}\right)\right|-\alpha_{i}^{t}g_{i}\left(u^{t}\right)=0. \end{array}$$

Substituting (22) in (21), it can be obtained

(23) $$\begin{array}{c} \left|g_{i}\left(u^{t+1}\right)\right|-\alpha_{i}^{t}g_{i}\left(u^{t+1}\right)\geq \left|g_{i}\left(u^{t}\right)\right|-\alpha_{i}^{t}g_{i}\left(u^{t}\right). \end{array}$$

According to the Step 3 in Algorithm 2 and the theory of SVD, for each iteration *t*, we have

(24) $$\begin{array}{c} \overset{N}{\underset{i=1}{\sum }}\alpha_{i}^{t}g_{i}\left(u^{t+1}\right)\geq \overset{N}{\underset{i=1}{\sum }}\alpha_{i}^{t}g_{i}\left(u^{t}\right). \end{array}$$

Combining (23) and (24) for every *i*, we have

(25) $$\begin{array}{c} \overset{N}{\underset{i=1}{\sum }}\left(\left|g_{i}\left(u^{t+1}\right)\right|-\alpha_{i}^{t}g_{i}\left(u^{t+1}\right)\right)\geq \overset{N}{\underset{i=1}{\sum }}\left(\left|g_{i}\left(u^{t}\right)\right|-\alpha_{i}^{t}g_{i}\left(u^{t}\right)\right)\\ \Rightarrow \overset{N}{\underset{i=1}{\sum }}\left|g_{i}\left(u^{t+1}\right)\right|\geq \overset{N}{\underset{i=1}{\sum }}\left|g_{i}\left(u^{t}\right)\right|, \end{array}$$

which means that Algorithm 2 is monotonically increasing. Additionally, considering that objective function (14) of KECA-L1 has an upper bound within the limited iterations, the KECA-L1 procedure will converge.

### 3.3. The Semisupervised Classifier

Jenssen [26] established a semisupervised learning (SSL) algorithm for classification using KECA. This SSL-based classifier was trained by both labeled and unlabeled data to build the kernel matrix such that it can map the data to KFS appropriately [26]. Additionally, it is based on a general modelling scheme and applicable for other variants of KECA, such as OKECA and KECA-L1.

More specifically, we are given *N* pairs of training data {**x**_*i*_, *y*_*i*_}_*i*=1_^*N*^ with samples **x**_*i*_ ∈ *ℝ* ^*D*^ and the associated labels *y*_*i*_. In addition, there are *M* unlabeled data points for testing. Let **X**_*u*_=[**x**_*u*_^1^,…, **x**_*u*_^*M*^] and **X**_*l*_=[**x**_*l*_^1^,…, **x**_*l*_^*N*^] denote the testing data and training data without labels, respectively; thus, we can obtain an overall matrix **X**=[**X**_*u*_  **X**_*l*_]. Then we construct the kernel matrix **K** derived from **X** using (6), **K** ∈ *ℝ* ^(*N*+*M*)×(*N*+*M*)^, which plays as the input of Algorithm 2. After the iteration procedure of nongreedy L1-norm maximization, we obtain a projection of $X⟶\tilde{E}={\left[{\tilde{E}}_{u}{\tilde{E}}_{l}\right]}_{m\times \left(M+N\right)}$ onto *m* orthogonal axes, where ${\tilde{E}}_{u}=\left[{\tilde{e}}_{1}^{u},\ldots ,{\tilde{e}}_{M}^{u}\right]$ and ${\tilde{E}}_{l}=\left[{\tilde{e}}_{1}^{l},\ldots ,{\tilde{e}}_{N}^{l}\right]$. In other words, ${\tilde{e}}_{i}^{u}$ and ${\tilde{e}}_{j}^{l}$ are the low-dimensional representations of each testing data point **x**_*u*_^*i*^ and the training one **x**_*l*_^*j*^, respectively. Assume that **x**_*u*_^*∗*^ is an arbitrary data point to be tested. If it satisfies

(26) $$\begin{array}{c} {\left\|{\tilde{e}}_{u}^{*}-{\tilde{e}}_{l}^{j}\right\|}_{2}=\underset{h=1,\ldots ,N}{\min}{\left\|{\tilde{e}}_{u}^{*}-{\tilde{e}}_{l}^{h}\right\|}_{2}, \end{array}$$

then **x**_*u*_^*∗*^ is assigned to the same class with the *j*th data point of **X**_*l*_.

## 4. Experiments

This section shows the performance of the proposed KECA-L1 compared with the classical KECA [6] and OKECA [21] for real-world data classification using the SSL-based classifier illustrated in Section 3.3. Several recent techniques such as PCA-L1 [27] and KPCA-L1 [28] are also included for comparison. The rationale to select these methods is that previous studies related to DR found that they can produce impressive results [27–29]. We implement the experiments on a wide range of real-world datasets: (1) six different datasets from the University California Irvine (UCI) Machine Learning Repository (available at http://archive.ics.uci.edu/ml/datasets.html) and (2) 9 different software projects with 34 releases from the PROMISE data repository (available at http://openscience.us/repo). The MATLAB source code for running KECA and OKECA, uploaded by Izquierdo-Verdiguier et al. [21], is available at http://isp.uv.es/soft_feature.html. The coefficients set for PCA-L1 and KPCA-L1 is the same with [27, 28]. All of the experiments are all performed by MATLAB R2012a on a PC with Inter Core i5 CPU, 4 GB memory, and Windows 7 operating system.

### 4.1. Experiments on UCI Datasets

The experiments are conducted on six datasets from the UCI: the Inonosphere dataset is a binary classification problem of whether the radar signal can describe the structure of free electrons in the ionosphere or not; the Letter dataset is to assign each black-and-white rectangular pixel display to one of the 26 capital letters in the English alphabet; the Pendigits handles the recognition of pen-based handwritten digits; the Pima-Indians data set constitutes a clinical problem of diabetes diagnosis in patients from clinical variables; the WDBC dataset is another clinical problem for the diagnosis of breast cancer in malignant or benign classes; and the Wine dataset is the result of a chemical analysis of wines grown in the same region in Italy but derived from three different cultivars.  Table 1 shows the details of them. In the subsequent experiments, we just utilized the simplest linear classifier [30]. The theory of maximizing maximum likelihood (ML) [31] is selected as the rule for selecting bandwidth coefficient as suggested in [21].

**Figure 1:**
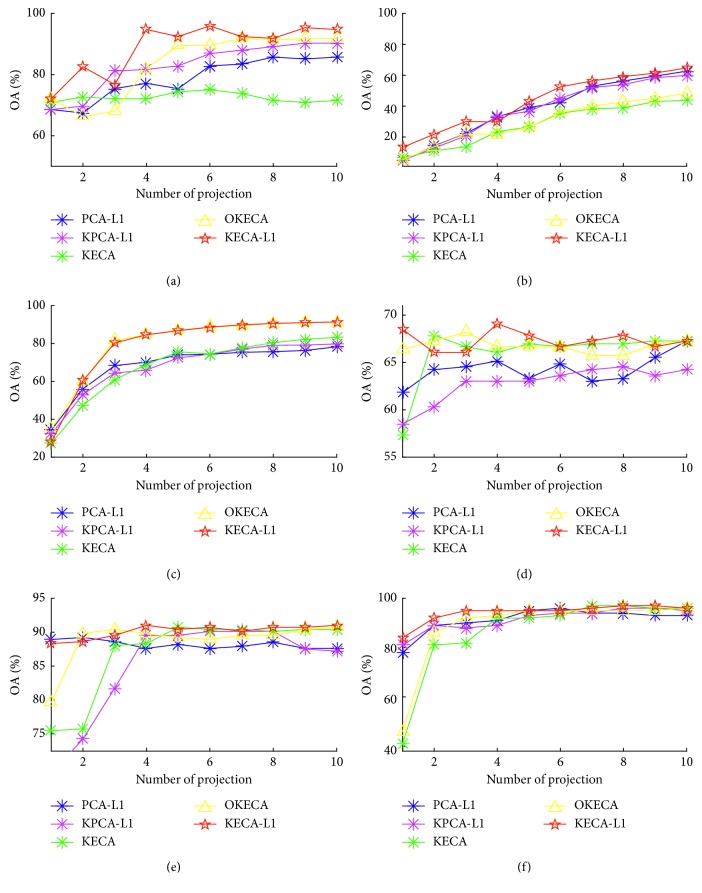
Overall accuracy obtained by the PCA-L1, KPCA-L1, KECA, OKECA, and KECA-L1 using different UCI databases with different numbers of extracted features. (a) Ionosphere, (b) Letter, (c) Pendigits, (d) Pima-Indians, (e) WDBC, and (f) Wine.

The implementation of KECA-L1 and other methods is repeated using all the selected datasets with respect to different numbers of components for 10 times. We have utilized the overall classification accuracy (OA) to evaluate the performance of different algorithms on the classification. OA is defined as the total number of samples correctly assigned in percentage terms, which is within [0,1] and indicates better quality with larger values.  Figure 1 presents the average OA curves obtained by the aforementioned algorithms for these six real datasets. It can be observed from Figure 1 that OKECA is superior to KECA, PCA-L1, and KPCA-L1 except for solving Letter issue. This is probably because DR performed by OKECA not only can reveal the structure related to the most Renyi entropy of the original data but also consider the rotational invariance property [21]. In addition, KECA-L1 outperforms the other methods besides of OKECA. This may be attributed to the robustness of L1-norm to outliers compared with that of the L2-norm. In Figure 1, OKECA seems to obtain nearly the same results with KECA-L1's. However, the average running time (in hours) of OKECA in the Pendigits is 37.384 times more than that of KECA-L1 1.339.

### 4.2. Experiments on Software Projects

In software engineering, it is usually difficult to test a software project completely and thoroughly with the limited resources [32]. Software defect prediction (SDP) may provide a relatively acceptable solution to this problem. It can allocate the limited test resources effectively by categorizing the software modules into two classes: nonfault-prone (NFP) or fault-prone (FP) according to 21 software metrics (Table 2).

This section aims to employ KECA-based methods to reduce the selected software data (Table 3) dimensions and then utilize the SSL-based classifier combined with the support vector machine [33] to classify each software module as NFP or FP. The bandwidth coefficient set is still restricted to the rule of ML. PCA-L1 and KPCA-L1 are involved as a benchmarking yardstick. There are 34 groups of tests for each release in Table 3. The most suitable releases [34] from different software projects are selected as training data. We evaluate the performance of different selected methods on SDP in terms of recall (R), precision (P), and F-measure (F) [35, 36]. The F-measure is defined as

(27) $$\begin{array}{c} F=\frac{2\times \text{precsion}\times \text{recall}}{\text{precsion}+\text{recall}}, \end{array}$$

where

(28) $$\begin{array}{c} \text{Precsion}=\frac{\text{TP}}{\text{TP}+\text{FP}}\text{,}\\ \text{Recall}=\frac{\text{TP}}{\text{TP}+\text{FN}}. \end{array}$$

In (28), FN (i.e., false negative) means that buggy classes are wrongly classified to be nonfaulty, while FP (i.e., false positive) means nonbuggy classes are wrongly classified to be faulty. TP (i.e., true positive) refer to correctly classified buggy classes [34]. Values of Recall, Precision, and F-measure range from 0 to 1 and higher values indicate better classification results.

**Figure 2:**
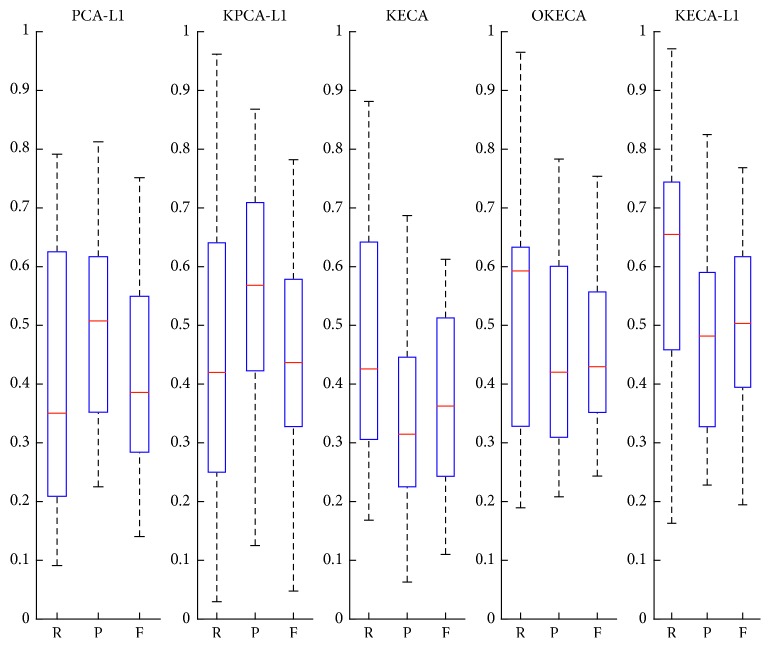
The standardized boxplots of the performance achieved by PCA-L1, KPCA-L1, KECA, OKECA, and KECA-L1, respectively. From the bottom to the top of a standardized box plot: minimum, first quartile, median, third quartile, and maximum.

Figure 2 shows the results using box-plot analysis. From Figure 2, considering the minimum, maximum, median, first quartile, and third quartile of the boxes, we find that KECA-L1 performs better than the other methods in general. Specifically, KECA-L1 can obtain acceptable results in experiments for SDP compared with the benchmarks proposed in Reference [34], since the median values of the boxes with respect to R and F are close to 0.7 and more than 0.5, respectively. On the contrary, not only KECA and OKECA but PCA-L1 and KPCA-L1 cannot meet these criteria. Therefore, all of the results validate the robustness of KECA-L1.

## 5. Conclusions

This paper proposes a new extension to the OKECA approach for dimensional reduction. The new method (i.e., KECA-L1) employs L1-norm and a rotation matrix to maximize information potential of the input data. In order to find the optimal entropic kernel components, motivated by Nie et al.'s algorithm [23], we design a nongreedy iterative process which has much faster convergence than OKECA's. Moreover, a general semisupervised learning algorithm has been established for classification using KECA-L1. Compared with several recently proposed KECA- and PCA-based approaches, this SSL-based classifier can remarkably promote the performance on real-world datasets classification and software defect prediction.

Although KECA-L1 has achieved impressive success on real examples, several problems still should be considered and solved in the future research. The efficiency of KECA-L1 has to be optimized for it is relatively time-consuming compared with most existing PCA-based methods. Additionally, the utilization of KECA-L1 is expected to appear in each pattern analysis algorithm previously based on PCA approaches.

## Figures and Tables

**Algorithm 1:**
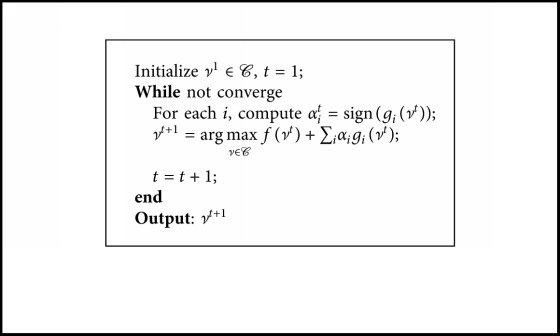
Fast iteration approach to solving the general L1-Norm maximization problem (3).

**Algorithm 2:**
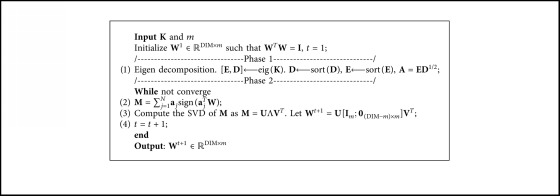
KECA-L1.

**Table 1:** UCI datasets description.

Database	*N*	DIM	*N*_c_	*N*_train_	*N*_test_
Ionosphere	351	33	2	30 × 2	175
Letter	20000	16	26	35 × 26	3870
Pendigits	10992	16	9	60 × 9	3500
Pima-Indians	768	8	2	100 × 2	325
WDBC	569	30	2	35 × 2	345
Wine	178	12	3	30 × 3	80

*N*: number of samples, DIM: number of dimensions, *N*_c_: number of classes, *N*_train_: number of training data, and *N*_test_: number of testing data.

**Table 2:** Descriptions of data attributes.

Attribute	Description
WMC	Weighted methods per class
AMC	Average method Complexity
AVG_CC	Mean values of methods in the same class
CA	Afferent couplings
CAM	Cohesion among methods of class
CBM	Coupling between Methods
CBO	Coupling between object classes
CE	Efferent couplings
DAM	Data access Metric
DIT	Depth of inheritance tree
IC	Inheritance Coupling
LCOM	Lack of cohesion in Methods
LCOM3	Normalized version of LCOM
LOC	Lines of code
MAX_CC	Maximum values of methods in the same class
MFA	Measure of function Abstraction
MOA	Measure of Aggregation
NOC	Number of Children
NPM	Number of public Methods
RFC	Response for a class
Bug	Number of bugs detected in the class

**Table 3:** Descriptions of software data.

Releases	#Classes	#FP	% FP
Ant-1.3	125	20	0.160
Ant-1.4	178	40	0.225
Ant-1.5	293	32	0.109
Ant-1.6	351	92	0.262
Ant-1.7	745	166	0.223
Camel-1.0	339	13	0.038
Camel-1.2	608	216	0.355
Camel-1.4	872	145	0.166
Camel-1.6	965	188	0.195
Ivy-1.1	111	63	0.568
Ivy-1.4	241	16	0.066
Ivy-2.0	352	40	0.114
Jedit-3.2	272	90	0.331
Jedit-4.0	306	75	0.245
Lucene-2.0	195	91	0.467
Lucene-2.2	247	144	0.583
Lucene-2.4	340	203	0.597
Poi-1.5	237	141	0.595
Poi-2.0	314	37	0.118
Poi-2.5	385	248	0.644
Poi-3.0	442	281	0.636
Synapse-1.0	157	16	0.102
Synapse-1.1	222	60	0.270
Synapse-1.2	256	86	0.336
Synapse-1.4	196	147	0.750
Synapse-1.5	214	142	0.664
Synapse-1.6	229	78	0.341
Xalan-2.4	723	110	0.152
Xalan-2.5	803	387	0.482
Xalan-2.6	885	411	0.464
Xerces-init	162	77	0.475
Xerces-1.2	440	71	0.161
Xerces-1.3	453	69	0.152
Xerces-1.4	588	437	0.743

## Data Availability

The data used to support the findings of this study are available from the corresponding author upon request.
