# Supplementary material for: Kernel Entropy Component Analysis with Nongreedy L1-Norm Maximization
Source: Comput Intell Neurosci. 2018 Oct 14;2018:6791683. doi: 10.1155/2018/6791683 (PMC6204191; doi:10.1155/2018/6791683)
Supplement: Supplementary Materials — The MATLAB toolbox of KECA-L1 is available. [file 6791683.f1.pdf]

Dear editor,

This text is the main parts of source code of this paper implemented in Matlab R2015a. For more detailed information, please run the file “Demo_Classification.m” in “KECA-L1(new).zip”. Thank you very much for your consideration.

Best wishes.

Yours sincerely,

Haijin Ji

```
%%%%%%%%%%%%        Demo_Classification.m       %%%%%%%%%%%%%%%
%%% Demo that predicts the label arrays of ionosphere data using KECA and L1-KECA
%%% ionosphere data from the University California Irvine
%%% (UCI) Machine Learning Repository (http://archive.ics.uci.edu/ml/datasets.html)

clc;clear;
addpath(genpath('./tools'))                % including clusKECAL1.m and clusKECA.m
addpath(genpath('./methods'))              % including KECA_L1.m and keca.m
load('ionosphere.mat');
Data(:,2) = [];
Data(:,1) = [];
Data = Data';
Data1 = Data(1:32,:);

%%% estimating kernel width parameter
X = Data1;
ind = randperm(size(X,2));
dat1 = X(:,ind(1:round(size(X,2)*0.9)));
dat2 = X(:,ind(round(size(X,2)*0.9)+1:end));

SIGMAS = logspace(-2,2,25);
for ind_s =1:length(SIGMAS)

    n1sq = sum(dat1.^2);
    n1 = size(dat1,2);
    n2sq = sum(dat2.^2,1);
    n2 = size(dat2,2);
    D = (ones(n2,1)*n1sq)' + ones(n1,1)*n2sq -2*dat1'*dat2;

    K = (1/(SIGMAS(ind_s)*sqrt(2*pi)))*exp(-D/(2*SIGMAS(ind_s)^2));

    MLE(ind_s) = sum(log(sum(K)./size(K,2)));
end

% maximum likelihood
```

```
[~,ii] = max(MLE);
sigma = SIGMAS(ii);

%%% randomly selecting training and testing data
label = Data(33,:);
t1 = find(label==1);
t2 = find(label==2);
iii = t1(randperm(length(t1),30));
iii = [iii t2(randperm(length(t2),30))];
jjj = setdiff(1:351,iii);
jjj = jjj(randperm(351-60,172));

X1 = Data(1:32,iii);%training data
Y1 = label(iii);
Y3 = label(jjj);
X3 = Data(1:32,jjj);%testing data


% extracted componets
        [Phi3,Phi4] = clusKECA(X3',X1',sigma);
        for d=1:10
            [Phi1,Phi2] = clusKECAL1(X3',X1',sigma,d);
            testECA = Phi1(:,1:d);
            trainECA = Phi2(:,1:d);
            class2 = classify(testECA,trainECA,Y1);
            keca1= sum(class2'==Y3)/length(Y3);

            testECA = Phi3(:,1:d);
            trainECA = Phi4(:,1:d);
            class2 = classify(testECA,trainECA,Y1);
            keca2= sum(class2'==Y3)/length(Y3);

            Res1(d) = keca1*100;
            Res2(d) = keca2*100;
        end
%% plot
figure
plot(1:length(Res1),Res1,'r-p','LineWidth',1)
hold on
plot(1:length(Res1),Res2,'g-*','LineWidth',1)
box off
axis([1 10 1 100])
legend('KECA-L1','KECA','Location','southeast')
xlabel('Number of Projections','FontSize',12,'FontWeight','bold','FontName','Times New Roman')
```

```
ylabel('OA (%)','FontSize',12,'FontWeight','bold','FontName','Times New Roman')

%%%%%%%%%%%%%%%    clusKECAL1.m   %%%%%%%%%%%%%%%%%%%%
% Compute the entropic components of KECA-L1 method for classification
%
% [Phi1,Phi2] = clusKECAL1(test_data,train_data,sigma)
%
% inputs:
%
% - test_data:        (Size: [NxD]!) testing data matrix, each row is one observation, each column
is one feature
% - train_data:       (Size: [NxD]!) training data matrix, each row is one observation, each column
is one feature
% - sigma:            kernel width parameter
% - m:       the dimension of the projected data
% outputs:
%
% - Phi1:             dimensionanlity-reduced testing data;
% - Phi2:             dimensionanlity-reduced training data;
%
% Copyright (c) 2018   Haijin Ji, Song Huang et al.
function [Phi1,Phi2] = clusKECAL1(test_data,train_data,sigma,m)

M = size(test_data,1);
% Computes the kernel matrix
data = [test_data;train_data];
[K] = kernel(data,sigma);

% Computes B of KECA_L1
B = KECA_L1(K,m);

% Performs the kernel ECA mapping
Phi1 = B(1:M,:);
Phi2 = B(M+1:end,:);



%% Computing kernel matrix
function [K] = kernel(data,sigma)

    X = data';
    n1sq = sum(X.^2);
    n1 = size(X,2);
    D = (ones(n1,1)*n1sq)' + ones(n1,1)*n1sq -2*X'*X;
```

```
    K = (1/size(X,1))*(1/(sigma*sqrt(2*pi)))*exp(-D/(2*sigma^2));

%%%%%%%%%%%%%%%%%%    clusKECA.m    %%%%%%%%%%%%%%%%%%%%%%%%%%%%
% Compute the entropic components of KECA method for classification
%
% [Phi1,Phi2] = clusKECA(test_data,train_data,sigma)
%
% inputs:
%
% - test_data:      testing data matrix, each row is one observation, each column is one feature
% - train_data:     training data matrix, each row is one observation, each column is one feature
% - sigma:          kernel width parameter
% outputs:
%
% - Phi1:           dimensionanlity-reduced testing data;
% - Phi2:           dimensionanlity-reduced training data;
%
% Copyright (c) 2018    Chengzu Bai, Ren Zhang, Zeshui Xu

function [Phi1,Phi2] = clusKECA(test_data,train_data,sigma)

M = size(test_data,1);
% Computes the kernel matrix
data = [test_data;train_data];
[K] = kernel(data,sigma);

% Computes B of KECA
B = keca(K);

% Performs the kernel ECA mapping
Phi1 = B(1:M,:);
Phi2 = B(M+1:end,:);

%% Computing kernel matrix
function [K] = kernel(data,sigma)

    X = data';
    n1sq = sum(X.^2);
    n1 = size(X,2);
    D = (ones(n1,1)*n1sq)' + ones(n1,1)*n1sq -2*X'*X;

    K = (1/size(X,1))*(1/(sigma*sqrt(2*pi)))*exp(-D/(2*sigma^2));
```

```
%%%%%%%%%%%%%%%    KECA_L1.m    %%%%%%%%%%%%%%%%%%%%%
% KECA_L1 Compute the entropic components of KECA-L1 method.
%
% [BB,U]=KECA_L1(K,m)
%
% inputs:
%
% - K:          Kernel matrix
% - m:          the dimension of the projected data
%
% outputs:
%
% - BB:                 KECA-L1 features in colums;
% - W:                  Extra rotation matrix
%
% Copyright (c) 2018    Haijin Ji, Song Huang et al.
%
function [BB,W]=KECA_L1(K,m)


        %% Step 1: Eigendecomposition
[DIM,N] = size(K);
D = -5;
kk = 0;
% K = K';
while(min(diag(D))<0 || sum(abs(imag(diag(D))))>0)
      [V,D] = eigs(full(K + kk*eye(size(K,1))),DIM);
      kk = kk + max([sum(abs(imag(diag(D)))) abs(min(diag(D)))]);
end

[~,iii] = sort(diag(D));
V = V(:,iii);
dD = diag(D);
D = diag(dD(iii));
Q = (D.^0.5)*V';
% Q = Q';

% U=zeros(DIM,m);
% for i=1:m

        %% Step 2: Initiation
        Temp=rand(DIM,m);
        W=orth(Temp);
```

```
	%% Step 3: Polarity Check
	P=zeros(m,N); % Polarity function
	W0=zeros(DIM,m);

	%% Step 4: Convergence check
	while(W0~=W)
%			bool=1;
%			while(bool)
%				bool=0;
				for i=1:N
					clear Temp
					Temp = W'*Q(:,i);
					Temp(find(Temp>=0)) = 1;
					Temp(find(Temp<0)) = -1;
					P(:,i) = Temp;
				end
%			end

	%% Step 5: SVD
	  M = zeros(N,m);
		for i=1:N
			Temp1 = P(:,i);
			M = M + Q(:,i)*Temp1';
		end
	[U,~,V] = svd(M);
	clear W0
%	   clear W
	W0 = W;
	W = U*[eye(m,m);zeros(DIM-m,m)]*V';
	end
% end
% BB = W'*(D.^0.5)*V';
% BB = V*D.^0.5*W;
BB = W'*Q;
% BB = abs(BB');
BB = -BB';
valores_keca_2 = sum(abs(BB));
[~,iii] = sort(valores_keca_2,'descend');
BB = BB(:,iii);
% %
% % % MMM = MMM(iii);
% W = W(:,iii);

End
```

```
%%%%%%%%%%%%%%%%    keca.m    %%%%%%%%%%%%%%%%%%%%
% KECA Computes the principal components of the KECA method
%
% Input:    - K: kernel matrix. (Size: [NxN])
%
% Output: - V: KECA eigenvectors. (Size: [NxN])
%              - D: KECA eigenvalues. (Size: [NxN])
%
% Copyright (c) 2013    Emma Izquierdo-Verdiguier, Valero Laparra,
%                                  Robert Jenssen, Luis G 贸 mez-Chova, and Gustavo Camps-Valls
%
%                                  emma.izquierdo@uv.es, http://isp.uv.es
%

function [V,D]=keca(K)

    D = -5;
    kk = 0;
    OPTIONS.disp = 0;
    while(min(diag(D))<0 || sum(abs(imag(diag(D))))>0)
        [V D] = eigs(K + kk*eye(size(K,1)),size(K,1));
        kk = kk + max([sum(abs(imag(diag(D)))) abs(min(diag(D)))]);
    end

    valores_keca = sum((V*(D.^0.5))).^2;

    [valores_keca ind_keca] = sort(valores_keca,'descend');

    V=V(:,ind_keca);
    D=D(ind_keca,ind_keca);
```
